# Effects of Maternal Stress on Breast Milk Production and the Microbiota of Very Premature Infants

**DOI:** 10.3390/nu15184006

**Published:** 2023-09-16

**Authors:** María del Carmen Fernández-Tuñas, Alejandro Pérez-Muñuzuri, Rocío Trastoy-Pena, María Luisa Pérez del Molino, María L. Couce

**Affiliations:** 1 Department of Neonatology, University Clinical Hospital of Santiago de Compostela, 15706 Santiago de Compostela, Spain; carmen.fernandez.tunas@sergas.es; 2 IDIS-Health Research Institute of Santiago de Compostela, 15706 Santiago de Compostela, Spain; 3 Primary Care Interventions to Prevent Maternal and Child Chronic Diseases of Perinatal and Developmental Origin (RICORS-SAMID), Carlos III Health Institute, 5 Monforte de Lemos Av., 28029 Madrid, Spain; 4 Faculty of Medicine, University of Santiago de Compostela, 15706 Santiago de Compostela, Spain; 5 Department of Microbiology, University Hospital of Santiago de Compostela, Santiago de Compostela University, 15706 A Coruña, Spain; rocio.trastoy.pena@sergas.es (R.T.-P.); maria.luisa.perez.del.molino.bernal@sergas.es (M.L.P.d.M.)

**Keywords:** fecal calprotectin, maternal stress, microbiota, mother’s own milk, very preterm

## Abstract

Perinatal stress experienced by mothers of very premature newborns may influence the mother’s milk and the infant’s intestinal microbiota. This prospective study of mothers of very preterm infants fed with mother’s own milk (MOM) was carried out in a tertiary hospital over a 2-year period. The assessment of maternal stress in 45 mothers of 52 very preterm newborns using the parental stress scale (PSS:NICU) revealed an inverse relationship between stress and MOM production in the first days of life (*p* = 0.012). The greatest contributor to stress was the one related to the establishment of a mother–child bond. Maternal stress was lower in mothers in whom the kangaroo method was established early (*p* = 0.011) and in those with a higher educational level (*p* = 0.032). Levels of fecal calprotectin (FC) decreased with the passage of days and were directly correlated with birthweight (*p* = 0.044). FC levels 7 days post-delivery were lower in newborns that received postnatal antibiotics (*p* = 0.027). High levels of maternal stress resulted in progressive decreases and increases in the proportions of Firmicutes and Proteobacteria species, respectively, over 15 days post-delivery, both in MOM and in fecal samples from premature newborns. These findings underscore the importance of recognizing and appropriately managing maternal stress in neonatal units, given its marked influence on both the microbiota of maternal milk and the intestinal microbiota of premature newborns.

## 1. Introduction

Transmission of maternal bacteria to the intestine of a newborn through breast milk has been widely demonstrated, influencing the development of the intestinal microbiota of a newborn. Mother’s own milk (MOM) is the gold standard nutritional source during the first months of life in both full-term and premature infants. Milk from mothers of premature newborns is adapted to the moment of the infants’ birth, containing higher levels of immunological and anti-inflammatory factors (e.g., lactoferrin), and of protein, sodium, fat, and minerals such as calcium or phosphorus. Premature newborns absorb a very high percentage of lipids from MOM (90%), with a very important contribution from long-chain polyunsaturated fatty acids (LC-PUFA), such as docosahexaenoic acid and arachidonic acid, wherein energy intake from breast milk is higher than in a full-term newborns (58–70 Kcal/dL vs. 48–64 Kcal/dL) [1,2,3]. Very preterm infants fed with mother’s own milk (MOM) have lower rates of neonatal morbidity and improved long-term metabolic and neurocognitive outcomes [4,5,6]. Furthermore, MOM has large amounts of circulating microRNAs, some of which are of great relevance in the immunoregulation of a newborn’s immune system. Health teams working in neonatal intensive care units (NICUs) should provide support to the mothers of premature newborns to facilitate breastfeeding establishment and maintenance, her presence for the newborn, and establish the kangaroo method as early as possible, which favors a reduction in maternal stress and facilitates the production of breast milk [7,8,9,10,11].

The vast majority of premature newborns initially require donated human milk (DHM) when MOM is still not enough to cover a newborn’s needs, probably due to maternal stress from the premature birth, separation of the mother from her baby, or concern about the clinical status of the child. In these circumstances, breast milk establishment and maintenance will be an important challenge. DHM is pasteurized in the donated human milk banks. The caloric or macronutrient content is not altered by this process, although it can variably affect the biologically active components. The potential mechanisms by which DHM reduces necrotizing enterocolitis (NEC) [12] include reduced exposure to bovine antigens and the effects of functional components such as lactoferrin and human milk oligosaccharides, which may act positively on gut microbiota [13,14,15].

The mother’s microbiota colonizes the child from the intestinal bacteria during pregnancy with bacteria present in the birth canal, including the vaginal and perianal area, and bacteria from the skin and in MOM. This process favors adequate colonization by harmless microorganisms that protect a newborn from pathogens. Adequate and diverse colonization positively influences the immune, metabolic, cognitive, and sensory development of a newborn [16,17,18]. The intestinal microbiota of a newborn is very simple at birth and is influenced by the type of delivery, the use of antibiotics, or the type of diet, with the MOM being one of the first sources of bacteria. The bacterial phyla most frequently found in MOM includes *Firmicutes*, *Bacterioidetes*, *Actinobacteria*, and *Proteobacteria*, closely resembling the make-up of the mother’s intestinal bacteria. MOM also contains skin bacteria such as *Streptococcus* and *Staphylococcus*. Complex carbohydrates present in the MOM promote the development of *Bifidobacterium*, which are the dominant species in newborn’s intestine fed with MOM, that delay the implantation of enterobacteria. Infants fed with artificial formula have higher levels of *Enterobacteria* and lower levels of *Bifidobacterium* [19,20,21,22,23]. 

There is a dearth of studies assessing the effect of maternal stress on the gut microbiota of mothers of very premature newborns; most studies of prenatal stress have been conducted in animal models. Stress is known to promote intestinal and vaginal dysbiosis in the mother, transmitting it to the newborn. Studies carried out in both humans and animals show different results about bacterial diversity in relation to maternal stress. A recently published study tells us about greater bacterial biodiversity in the children of mothers with a mild/moderate level of prenatal stress, which makes them better prepared to face extrauterine life [24]. Although rarely taken into account, maternal stress is an important factor, whose appearance and inadequate management could lead to a poor establishment of breastfeeding, or even alter the production or modify the composition of breast milk. We examined the association between postnatal maternal stress and the production and composition of breast milk through the process of bacterial colonization of the intestine of very premature infants [24,25,26,27,28,29].

## 2. Material and Methods 

### 2.1. Study Design 

This prospective observational study was carried out over a 2-year period, from 1 May 2019 to 30 April 2021, at the NICU of the level III Clinical University Hospital of Santiago de Compostela. The study included premature newborns, weighing less than 1500 g and/or less than 32 weeks of gestational age (GA) who were breastfed, and their mothers. The follow-up of both mothers and neonates spanned the first 15 days of the newborns’ life. The study was approved by the local ethics committee (registration code 2019/229) and data were collected in an encrypted manner in a database.

### 2.2. Study Population

The study population consisted of 52 premature newborns with a GA ≤32 weeks and/or bodyweight ≤1500 g, and their 45 mothers, who provided written informed consent (IC) and breastfed their newborns (Supplementary Figure S1). The following data were collected:

- Sociodemographic data: mother’s age (≥35 years and <35 years); educational level (primary, secondary, higher); type of delivery (vaginal or cesarean section); GA; twinhood; newborn sex and bodyweight.
- Data on the clinical status of premature newborns: respiratory support (invasive mechanical ventilation [IMV], non-invasive mechanical ventilation [NIMV]) required or not; hemodynamically significant patent ductus arteriosus; sepsis; antibiotics received by newborn; antibiotics received by mother prior to delivery; days of parenteral nutrition; complete enteral nutrition; degree of feeding tolerance; start date of milk fortification; and evolution of newborn’s bodyweight.
- Initiation of kangaroo method: early (from birth to ≤4 days), intermediate (5–7 days), or late (≥8 days).
- Exposure to stress in mothers of premature newborns, measured using the parental stress scale (PSS:NICU).
- Breast milk parameters, determined by nutritional analysis and study of microbiota.
- Fecal calprotectin (FC) and microbiota, evaluated in fecal samples from premature newborns.

### 2.3. Inclusion and Exclusion Criteria

The following inclusion criteria were applied: all premature newborns ≤32 weeks GA and/or ≤1500 g body weight and their mothers, who specified the intention of breastfeeding and provided written IC to participate in the study. The exclusion criteria were as follows: newborns whose participation in the study was not compatible due to their very serious pathologies that did not make it possible to receive MOM; mothers who intend to give their own milk but abandoned to breastfeed in the 15 first days of life.

### 2.4. Outcome Measures

#### 2.4.1. Anthropometric Assessment

Weight was measured accurate to the nearest 10 g. Measurements were taken by the same researcher in duplicate to minimize intra-observer bias. Weight percentiles were calculated using the Fenton scale.

#### 2.4.2. Parental Stress Scale

To quantify stress in mothers of preterm infants, the PSS:NICU [30,31,32,33] was used. This consists of 5 subscales: (i) visual and auditory stimuli (VAS) in the NICU which is the stress generated by what they see and hear; (ii) physical appearance and behavior of the newborn (PAB), which is the stress generated by the physical appearance and behavior of the newborn; (iii) mother’s relationship with the newborn and maternal role (MR), defined as the stress caused by the individual perception of her role as mother and the establishment of the maternal bond; (iv) communication with health personnel (CWS), which is the stress generated by the relationship with the health personnel; (v) common stress (CS) defined as the individual perception of stress to which the mother is subjected. The complete scale consists of a total of 46 items, each of which is rated by the user using a 5-point Likert scale, where 1 corresponds to no stress and 5 to extremely stressful. The overall score of the 46 items is weighted to obtain a total stress (TS) rating. The total stress experienced by the mother was defined as high or low depending on whether it was above or below the median value on 3, 7, and 15 days after birth of the newborn.

#### 2.4.3. Nutritional Analysis of Macronutrients in MOM 

A 5-mL sample of fresh MOM was collected on days 3, 7, and 15 after the birth of the newborn and its nutritional content (proteins, fats, and carbohydrates) was analyzed using the human milk analyzer with FTIR technology (MilkoSkan^TM^ Mars, FOSS, Hilleroed, Denmark) [34]. MOM samples were collected directly from milk expressed by the mother in the morning when she attended the neonatal intensive care unit (NICU) using a breast pump. MOM that is extracted manually can have a higher fat content than milk extracted using a breast pump [35]. This was taken into account when collecting the samples, and the same process was used to collect all samples to avoid variation. MOM was collected in a sterile bottle, homogenized by shaking it several times, and heated in a water bath for immediate analysis. The analyzer used for the measurement underwent daily calibration before carrying out the macronutrient analysis.

#### 2.4.4. FC 

Stool samples for FC analysis were collected from the newborn’s diaper on days 3, 7, and 15 days after birth, and the sample immediately stored in a refrigerator for subsequent analysis by solid phase microplate enzyme immunoassay (ELISA).

#### 2.4.5. Analysis of Microbiota in Newborn Stool and MOM

Stool samples were collected directly from the diaper using a spatula and placed in a sterile recipient. To collect MOM samples, the nipple area of the breast was washed with soap and water and approximately 1 mL of milk was extracted using a breast pump and collected in a sterile plastic jar. The samples were frozen at −80 °C in tubes with screw caps for subsequent processing and analysis. Nucleic acid extraction was carried out using the Maxwell^®^ (San Diego, CA, USA) RSC instrument with the Maxwell^®^ RSC PureFood GMO and Authentication kit. Once genomic material was obtained, DNA was quantified using the Qubit DNA Assay (Thermo Fisher^®^, Waltham, MA, USA). The microbiota was analyzed using the MiSeq sequencer to carry out the 16 s microbiome protocol. The results were analyzed using Illumina BaseSpace together with the 16S Metagenomics application [36,37,38]. 

The Shannon index was used as an indicator to quantify biodiversity. A value of zero indicated the presence of a single species: the higher the index, the greater the diversity of species [39,40]. 

### 2.5. Data Collection

Data and samples for analysis were collected from mothers and newborns at birth and on days 3, 7, and 15 after birth. 

At birth, an interview was carried out with the mother where consent to participate in the study was obtained and the sociodemographic data to be analyzed were recorded. At this time, the mother was instructed on how to initiate breastfeeding, how to obtain milk samples, as well as recording of the amount of milk obtained throughout the 15 days of the study. Also, at that time the stress scale that was going to be used and how the data should be recorded was explained.

The analysis of breast milk was carried out with fresh samples using a 5 mL aliquot. Stool samples were taken directly from the diaper on days 3, 7, and 15 of life. They were stored and processed as previously indicated.

The rest of the clinical variables, such as the start of kangaroo care or the need for antibiotics, were noted in an anonymized database.

### 2.6. Statistical Analyses

Data were analyzed using the statistical package IBM SPSS Statistics version 20.0. The normality of the quantitative variables was assessed using the Shapiro–Wilk test. Variables were mostly non-parametric and are presented as the median and interquartile range.

Statistical analyses were carried out using the non-parametric Mann–Whitney U test for quantitative variables, the chi-squared test for qualitative variables, and the non-parametric Kruskal–Wallis test for comparison of three or more variables. The possible relationship between variables was studied using the Spearman correlation test or through univariate or multivariate linear regression studies. 

In all the comparisons of variables, statistical significance was set at *p* ≤ 0.05.

## 3. Results

### 3.1. Characteristics of the Study Participants

Table 1 and Table 2 show the data for the participating mothers and their infants. The study population consisted of 45 mothers and 52 very preterm newborns (24 females and 28 males) who met the inclusion criteria for the study. In newborns, there were no significant differences in sex distribution between groups. The mean percentile of weight decreased from P33 at birth to P15 at 3 days of life, after which bodyweight increased on the 15th percentile. Implementation of the kangaroo method was early or intermediate in 69.23% (36/52) of cases. Tolerance to enteral feeding among neonates was generally good, with complete enteral nutrition established at 15 days of life in 92.31% (48/52) of newborns. 

Median FC levels decreased with age and were higher at 7 days of life in newborns who did not receive antibiotics (*p* = 0.027) and newborns weighing <1000 g at birth (*p* = 0.044). 

Non significant differences were found depending on the respiratory, hemodynamic, or infectious variables in relation to the level of maternal stress.

**Figure 1:**
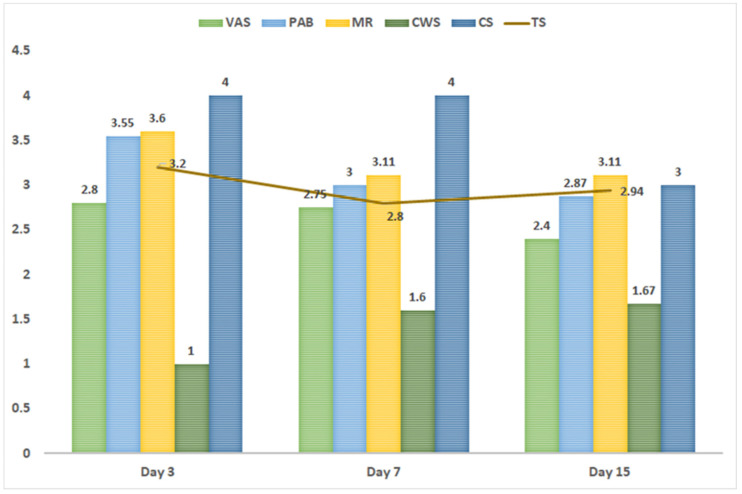
Stress scale scores for mothers on days 3, 7, and 15 post-delivery. CS, common stress; CWS, communication with healthcare team; d, day; MR, maternal role; PAB, physical appearance and behavior; TS, total stress; VAS, visual and auditory stimuli.

Common stress (CS) was 4 at days 3 and 7, indicating a very stressful situation, and decreased to 3 at day 15. Analysis of the individual subscales showed the highest stress scores for the MR subscale (Figure 1).

Table 3 shows significant associations between stress scores and other mother or newborn-related variables. 

The earlier the kangaroo method was established, the greater the reduction in stress was. Stress levels among mothers were very high over the first 3 days post-delivery, while MOM production over this period was very low. On day 7 post-delivery, stress levels decreased, and milk production increased, and continued to do so over the following days, although stress levels increased slightly. 

Stress management was poorer in the group of mothers that had completed only primary education versus those that had completed secondary or higher education. In the latter group, stress levels decreased sooner after delivery, with significant decreases observed at 7 and 15 versus 3 days (Table 3).

We have also defined stress as low or high according to the median value at each day, 3.2 at 3 days, 2.8 at 7 days, and 2.94 at 15 days. We studied many variables according to this definition (Table 4).

Mothers presented higher stress when the gestational age was lower, with no influence observed by age or type of delivery. Twinhood, on the other hand, seems to be a protective factor against stress. The volume of breast milk was greater in mothers with less stress. The results were not always significant, but a clear trend was observed.

### 3.2. Macronutrient Content in MOM

As expected, median levels of MOM fats and carbohydrates increased over time. Lipid levels were 1.79 g/dL, 3.16 g/dL, and 3.27 g/dL at 3, 7, and 15 days, respectively, and carbohydrates levels were 6.55 g/dL, 6.85 g/dL, and 7.19 g/dL at 3, 7, and 15 days, respectively. Protein levels had a slight decrease over time and were 1.4 g/dL, 1.3 g/dL, and 1.2 g/dL at 3, 7, and 15 days, respectively. We observed no association between the levels of macronutrients and maternal stress during the study period.

### 3.3. Microbiota in MOM and Neonatal Stool Samples

**Figure 2:**
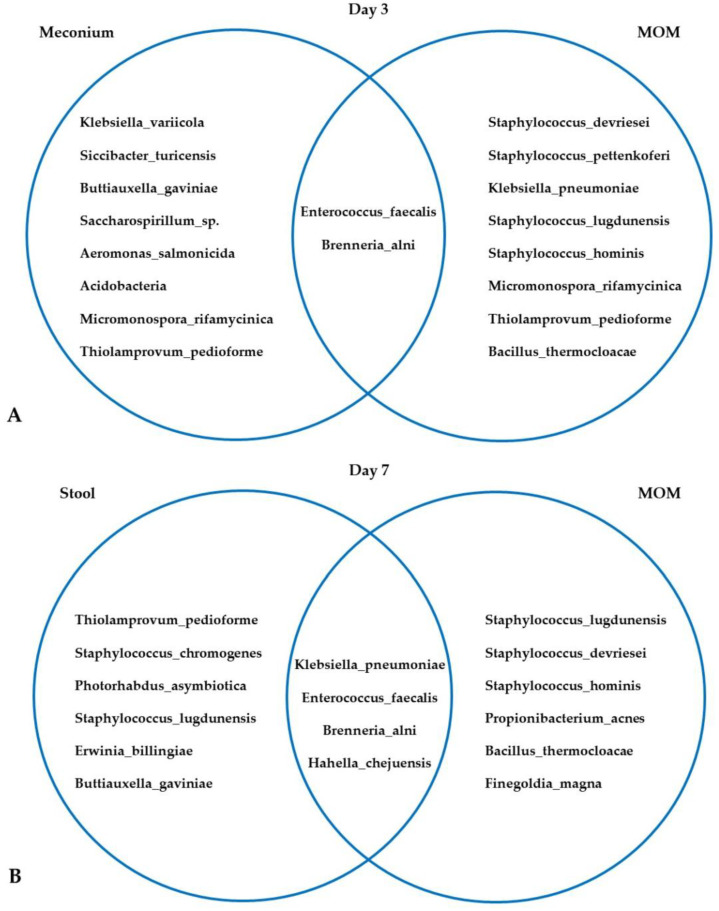

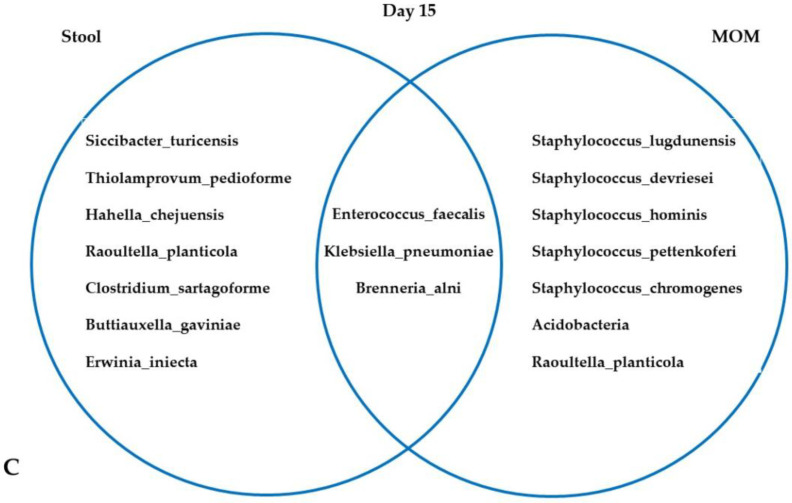
Microbiota of MOM and neonatal stool samples collected at days 3 (**A**), 7 (**B**), and 15 (**C**) post-delivery.

The Venn diagrams in Figure 2 depict the microbiota of MOM and neonatal stool samples collected at days 3, 7, and 15 post-delivery.

The bacteria detected in MOM samples included microorganisms commonly found on the skin *(Staphylococcus*, *Streptococcus*, *Bacillus*) and in the intestinal microbiota (*Klebsiella*, *Clostridium,* or *Enterococcus*). Bacteria found in neonatal fecal samples primarily consisted of species commonly found in the intestinal microbiota (*Clostridium*, *Enterobacter*, *Enterococcus*, *Klebsiella*). We investigated the association between microbial diversity in MOM and neonatal stool samples with maternal stress at days 3, 7, and 15 post-delivery (Table 5). Although the results were not statistically significant, a trend towards greater microbial diversity (i.e., a higher Shannon index) was observed in milk from mothers with continually decreasing maternal stress over the study. The pattern observed for fecal samples was more erratic, although we observed a similar trend to that of breast milk at 15 days.

The proportion of Proteobacteria, Firmicutes, Actinobacteria, and Bacteroidetes was analyzed in the MOM and neonatal fecal samples 3, 7, and 15 days post-delivery (Table 6).

Although the effect was not significant, we observed a greater proportion of Proteobacteria in neonatal fecal samples. Species of the phylum Firmicutes predominated in MOM. In milk samples from high-stress mothers, we observed a trend over the course of the study period towards decreasing proportions of Firmicutes (87.5%, 62.5%, and 55.6% on days 3, 7, and 15, respectively) and increasing proportions of Proteobacteria (12.5%, 37.5%, and 44.4% on days 3, 7, and 15, respectively). The opposite effect was observed in MOM samples from low-stress mothers (Table 7).

We observed a trend towards greater biodiversity in stool samples collected 7 days post-delivery from neonates who received complete enteral nutrition. Relative proportions of bacteria from the phyla Proteobacteria, Firmicutes, Actinobacteria, and Bacteroidetes (52.94%, 35.29%, 5.88%, 5.88%, respectively) were higher in neonates who received complete enteral nutrition compared to those who did not. Indeed, in the latter group, bacteria from the phyla Actinobacteria and Bacteroidetes were completely absent. 

## 4. Discussion

This study of very premature newborns and their mothers describes the negative effects of maternal stress on MOM production. It also shows a trend in the bacterial biodiversity of both milk and the neonatal intestinal microbiota depending on maternal stress, although it indicates that stress does not affect the macronutrient composition of milk. 

### 4.1. Parental Stress and MOM

Parental stress, if improperly managed, can lead to states of anxiety and depression in mothers, with significant negative impacts at both the personal and family level. High levels of parental stress following the birth of a very premature child can result in suboptimal bonding between mother and child poses a risk factor for poor behavioral and social development in childhood [3,7,8,9]. In our study population, the total stress in mothers of preterm infants decreased between 3 and 7 days post-delivery, subsequently increasing slightly at 15 days, possibly coinciding with the appearance of prematurity-associated complications in the newborn. In line with previously reported findings [33], the stress subscale for which the highest scores were observed was MR, with mean scores of 3.6 (day 3), 3.11 (day 7), and 3.11 (day 15). This subscale assesses stress related to the mother–child bond, including stress resulting from mother–child separation. It should also be noted that this study was carried out during the COVID-19 pandemic, and the resulting impact on hospital care in particular and society in general may have contributed to additional stress among mothers of premature newborns [10,41]. 

Situations of stress lead to increases in cortisol, adrenaline, dopamine, and other mediators of stress, as well as inhibition of prolactin and oxytocin, hormones central to the production and secretion of milk [28,42]. We observed a statistically significant inverse relationship between stress and milk production 3 days post-delivery. However, this effect was not significant at 7 or 15 days post-delivery, possibly because the initial stress caused by preterm labor decreased, and the mothers focused on breastfeeding their newborn, which in turn has a stress-reducing effect as it favors the release of hormones such as oxytocin [28]. We show that maternal stress was significantly reduced if the kangaroo method was established early (≤4 days post-delivery). Provision of psychological and emotional support to mothers of premature newborns should be implemented in all NICUs.

Our findings indicate better stress management in mothers with a higher educational level, possibly because they have access to greater social and emotional support, which allows them to better manage their stress levels [43].

In line with the results of other studies [44,45], maternal stress did not alter the macronutrient content of MOM [44,45]. Other authors have reported that in conditions of acute stress, in which cortisol levels increase, milk contains lower levels of carbohydrates and higher levels of fat, while in chronic stress conditions, fat levels in milk decrease [46].

### 4.2. FC, Microbiota, MOM, and Maternal Stress

FC is a protein that reflects the migration of neutrophils to the intestinal lumen and can serve as a biomarker of intestinal inflammation in adults, although in children conclusive FC values to establish a reference range are lacking [47,48]. FC levels appear to be linked to the maturity of the digestive system, resulting in higher levels in full-term and preterm newborns than in adults but with similar values between preterm and term newborns [49]. Multiple factors could explain the elevation of FC in the first days of life, including the intrauterine environment, immaturity of the intestine, possible hypoxic-ischemic damage to the intestinal mucosa, and even bacterial colonization of the intestine [50]. FC levels decrease over the following days, often due to the antibiotic treatment that preterm newborns undergo, which may delay intestinal colonization. When enteral feeding is established, FC concentrations rise again, and subsequently decrease within a few days in the absence of any pathology [51,52]. It is difficult to specify FC levels that are indicative of intestinal disease in premature infants, owing to the marked variability in this parameter, which is influenced by multiple factors. However, research continues to investigate how FC could serve as a marker to facilitate the detection of potentially fatal gastrointestinal disorders in premature newborns, such as NEC.

Bacteria found in the MOM microbiota included skin microorganisms (*Staphylococcus*, *Streptococcus*, *Bacillus*) and bacteria present in the intestinal microbiota (*Klebsiella*, *Clostridium*, or *Enterococcus*), while those found in newborn fecal samples largely corresponded to the intestinal microbiota (*Clostridium*, *Enterobacter*, *Enterococcus*, *Klebsiella*). The species that predominated in newborn fecal samples were *Enterococcus faecalis*, *E. coli*, *Klebsiella* spp. and *Staphylococcus* spp., with *Lactobacillus* and *Bifidobacteria* detected in small amounts. Prenatal or postnatal stress can also influence the newborn intestinal microbiota, and it is also likely that individual genetic factors modulate the effects of this stress [19,20,21,22,23].

The present study describes the effects of maternal stress on the diversity of microorganisms in MOM, and points to a trend towards reduced biodiversity with a lower Shannon index, in situations of greater stress. However, at days 3 and 7 post-delivery, when maternal stress levels were higher, microbial diversity in neonatal fecal samples was increased. This may be explained by intestinal dysbiosis resulting from stress, which allows bacteria not typically found in the normal microbiota to proliferate abnormally, hence increasing diversity. In infants born to mothers with higher stress levels from days 3 to 15, we observed a progressive increase in the proportion of Proteobacteria species (33.3%, 40%, and 70% on days 3, 7, and 15, respectively), while the opposite effect was observed for Firmicutes species (66.7%, 40%, and 20% on days 3, 7, and 15, respectively). In milk from mothers who experienced high levels of stress, the proportion of Firmicutes species was greater than that of Proteobacteria species at all three timepoints studied, but this progressively decreased over time (87.5%, 62.5%, and 55.6%, respectively), while the proportion of Proteobacteria species increased (12.5%, 37.5%, and 44.4%, respectively).

Some limitations of the present study should be noted. Although most premature infants required small amounts of DHM during the first days of the study period, its impact on the establishment of the microbiota was not the objective of this study (the perceived amount of DHM was similar for each of the patients studied); instead, we focused on how maternal stress influenced the establishment of breastfeeding and its influence on the microbiota. The study was carried out during the COVID pandemic, which may have affected the reported outcomes; fear of contagion may have influenced perceived levels of maternal stress and decreased rates of breastfeeding. In some cases, it was impossible to obtain complete data for all the study participants. The study’s main strength is its analysis of how stress influences the establishment of correct breastfeeding and intestinal colonization in the premature newborn.

## 5. Conclusions

We show that maternal stress influences bacterial biodiversity in the milk of mothers and in fecal samples from their premature newborns with clear trends despite non-significant results, which were probably due to the study limitations. This, in turn, affects the establishment of a healthy intestinal microbiota, which is required for adequate development and health of the premature newborn. Maternal stress, particularly that relating to the role of the mother, is a variable that must be taken into account in NICUs to ensure appropriate control and management, thereby increasing the production of milk and the bacterial biodiversity of both milk and the intestinal microbiota of the newborn.

Measures must be established to control maternal stress during the perinatal period. Although certain independent social variables (e.g., higher educational level) favor better stress control, additional measures can be applied in the NICU; as shown here, early initiation of the kangaroo method allows for more rapid control of stress caused by the separation from their premature newborn.

## Figures and Tables

**Table 1:** Characteristics of nursing mothers and their infants (part I).

		At Birth
Mothers’ age (years) median (interquartile range)		35 (31.5–38)
Gestational age (w)		31 (29–32)
Prenatal antibiotics (n, %)		17, 37.8%
Educational level (n, %)	Primary studies	4, 9%
	Secondary studies	18, 40%
	Higher education	23, 51%
Type of delivery (n, %)	Vaginal	17, 33%
	Caesarean section	35, 67%
Twinhood (n, %)		8, 15.4%
Sex of newborn: M/F (n, %)		M (28, 56.8%) F (24, 46.2%)

F, female; M, male; n, number; y, years; w, week.

**Table 2:** Characteristics of nursing mothers and their infants (part II).

		Time (Days)															
		At Birth	1	2	3	4	5	6	7	8	9	10	11	12	13	14	15
Neonatal weight (g), median (interquartile range), and percentile		1375 (1012–1667) p33			1240 (927–1467) p15				1280 (990–1515) p12								1520 (1125–1755) p14
Neonatal antibiotics in first 48 h (n, %)		34, 65.4%															
Duration of neonatal antibiotic treatment									7 days								
Respiratory support (n, %)	Invasive				2, 3.8%				0, 0%								1, 1.92%
	Non-invasive				38, 73.1%				16, 30.8%								7, 13.5%
Significant patent ductus arteriosus (n, %)					2, 3.8%				3, 5.8%								3, 5.8%
Sepsis (n, %)					7, 13.5%				4, 7.7%								3, 5.8%
Nutrition (n, %)	Parenteral				51, 98.1%				24, 46.2%								3, 5.8%
	Well tolerated				46, 88.5%				51, 98.1%								50, 96.2%
	Complete enteral nutrition				4, 7.7%				39, 75%								48, 92.3%
	Fortification				0, 0%				19, 36.5%								45, 86.5%
Initiation of kangaroo method (n, %)	Early	16, 30.8%															
	Intermediate						20, 38.5%										
	Late									16, 30.8%							
FC (mcg/g stool), median (interquartile range)					70 (20.25–148.75)				26 (7.25–60.75)								42 (28–72.75)
FC levels (mcg/g stool) in newborns with vs. without antibiotics, median (interquartile range)					83.5 (22–168) vs. 64 (19–129)				17 (6–39) vs. 54 (17–94) *p* = 0.027								49 (28–73) vs. 38 (29–54)
FC (mcg/g stool) in stool samples according to body weight: <1000 g; 1001–1500 g; >1500 g, median (interquartile range)					122 (55–246.5); 70 (21–127); 21 (12.5–120.5) *p* = 0.044				24.5 (14.5–45); 21.5 (6–54); 42.5 (8–78.5)								40 (7.5–71.5); 43 (29.5–91.5); 47.5 (30–66.5)

FC, fecal calprotectin; g, grams; mcg, micrograms; n, number.

**Table 3:** Associations between maternal stress scores and other mother and newborn-related variables.

	Day 3			Day 7			Day 15	
Breastfeeding volume	TS	*p*	Breastfeeding volume	TS	*p*	Breastfeeding volume	TS	*p*
22.5 mL (5–61)	3.2 (2.52–3.64)	0.012	150 mL (45–340)	2.8 (2.3–3.87)	NS	275 mL (130–530)	2.94 (2–3.8)	NS
Initiation of Kangaroo method	Decrease stress	*p*	Initiation of Kangaroo method	Decrease stress	*p*	Initiation of Kangaroo method	Decrease stress	*p*
Early	−0.65 (−1.00–−0.38)	0.011	Intermediate	0.02 (−0.41–0.34)	NS	Late	−0.38 (−0.66–−0.12)	NS
TS with Primary Educational level	TS with Secondary and Higher Educational level	*p*	TS with Primary Educational level	TS with Secondary and Higher Educational level	*p*	TS with Primary Educational level	TS with Secondary and Higher Educational level	*p*
3.64 (2.72–4.01)	3.2 (2.4–3.73)	NS	4.43 (3.48–4.58)	2.79 (2.25–3.78)	0.032	4.3 (3.14–4.51)	2.89 (1.84–3.7)	0.04

Breastfeeding volumes and total stress scores (TS) are presented as the median and interquartile range. mL, milliliters; NS, not significant.

**Table 4:** Associations between low or high maternal stress and other mother and newborn-related variables.

		Day 3			Day 7			Day 15		
		Low Stress (<3.2)	High Stress (≥3.2)	*p*	Low Stress (<2.8)	High Stress (≥2.8)	*p*	Low Stress (<2.94)	High Stress (≥2.94)	*p*
Mothers’ age (y) median (interquartile range)		35 (29.5–38.5)	35 (33–36)	NS	36 (35–39)	33.5 (26–36)	0.03	36 (32–39)	34 (26–36)	NS
Gestational age (w) median (interquartile range)		31 (29–32)	30 (27–31)	NS	31 (30–32)	30 (26–31)	0.031	31 (29–32)	30 (27–31)	NS
Type of delivery	Vaginal	33.3%	66.7%	NS	31.2%	68.8%	NS	43.7%	56.3%	NS
	Caesarean section	57.7%	42.3%		59.2%	40.8%		53.8%	46.2%	
Twinhood (Yes)		57.2%	42.8%	NS	87.5%	12.5%	0.015	87.5%	12.5%	0.018
Initiation of kangaroo method	Early	50%	50%	NS	66.7%	33.3%	NS	58.3%	41.7%	NS
	Intermediate	52.9%	47.1%		47.1%	52.9%		43.7%	56.3%	
	Late	38.4%	61.6%		30.7%	69.3%		46.1%	53.9%	
MOM volume median (interquartile range)		40 mL (11–122.5)	20 mL (5–48)	0.046	180 mL (117.5–370)	82.5 mL (44–340)	NS	392.5 mL (197.5–550)	220 mL (85–440)	NS

mL, milliliters; NS, not significant; y, years; w, week.

**Table 5:** Shannon index and maternal stress during the study period.

		Day 3		Day 7		Day 15	
Stress		Low (<3.2)	High (≥3.2)	Low (<2.8)	High (≥2.8)	Low (<2.94)	High (≥2.94)
Shannon index	Feces	1 (0.48–1.86)	1.56 (1.19–1.65)	0.71 (0.31–1.24)	1.25 (0.76–1.36)	1.13 (0.77–1.35)	0.91 (0.33–1.08)
	MOM	0.99 (0.60–1.39)	0.76 (0.61–1.01)	0.75 (0.46–1.21)	0.65 (0.38–1.14)	1.19 (0.60–1.34)	0.99 (0.34–1.37)

p NS (not significant).

**Table 6:** Proportions of indicated phyla in MOM and neonatal fecal samples.

	Day 3		Day 7		Day 15	
	Meconium	MOM	Feces	MOM	Feces	MOM
Proteobacteria	62.5%	30.4%	50%	30%	71.4%	34.6%
Firmicutes	37.5%	69.6%	35%	70%	17.9%	65.4%
Actinobacteria	N/A	N/A	5%	N/A	3.6%	N/A
Bacteroidetes	N/A	N/A	5%	N/A	3.6%	N/A

N/A: nondetectable.

**Table 7:** Proportions of the indicated phyla in MOM and neonatal fecal samples, stratified according to maternal stress scores.

Phyla in MOM	Stress 3 Days		Stress 7 Days		Stress 15 Days	
	Low	High	Low	High	Low	High
Proteobacteria	41.7%	12.5%	16.7%	37.5%	26.7%	44.4%
Firmicutes	58.3%	87.5%	83.3%	62.5%	73.3%	55.6%
Phyla in feces						
Proteobacteria	75%	33.3%	66.7%	40%	90%	70%
Firmicutes	25%	66.7%	22.2%	40%	10%	20%
Actinobacteria	N/A	N/A	11.1%	N/A	N/A	N/A
Bacteroidetes	N/A	N/A	N/A	20%	N/A	10%

N/A: nondetectable.

## Data Availability

Not applicable.

## Supplementary Materials

The following supporting information can be downloaded at: https://www.mdpi.com/article/10.3390/nu15184006/s1, Figure S1: Flow chart depicting recruitment of the study cohort.
