# Supplementary figures and images for: Effects of Maternal Stress on Breast Milk Production and the Microbiota of Very Premature Infants
Source: Nutrients. 2023 Sep 16;15(18):4006. doi: 10.3390/nu15184006 (PMC10534677; doi:10.3390/nu15184006)

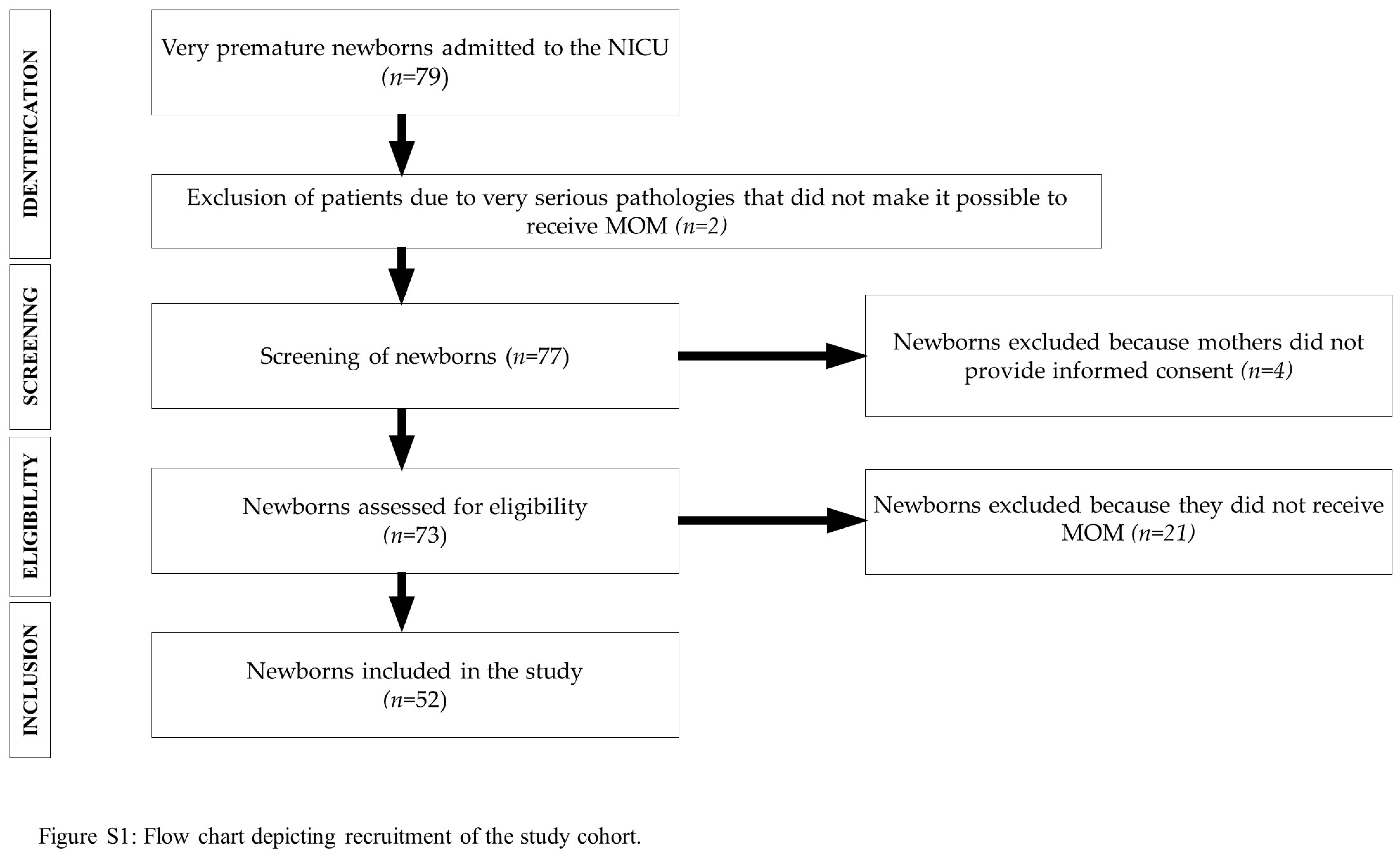

Supplement: Supplementary file 1 [file nutrients-15-04006-s001.zip › nutrients-2587159-supplementary.jpg]
